# Barriers to preventive care among US adults of multiple races eligible for type 2 diabetes screening: an observational study

**DOI:** 10.1186/s12889-025-24692-y

**Published:** 2026-01-10

**Authors:** Alain K. Koyama, Stephen Onufrak, Kai McKeever Bullard, Fang Xu, Michelle Papali’i, Yoshihisa Miyamoto, Ryan Saelee, Meda E. Pavkov

**Affiliations:** Division of Diabetes Translation, Centers for Disease Control and Prevention, 4770 Buford Hwy NE, MS-S107-3, Atlanta, GA 30341-3724 USA

**Keywords:** Diabetes, Epidemiology, Health care, Health disparities, Multiple race

## Abstract

**Background:**

Little is known about disparities in access to preventive care among people of multiple races, who are often aggregated into a single category, despite comprising a heterogeneous population. We therefore described the prevalence of barriers to preventive care for type 2 diabetes among US adults, by disaggregated subgroups of multiple races.

**Methods:**

In a pooled cross-sectional study of adults eligible for type 2 diabetes screening from the Behavioral Risk Factor Surveillance System (2013–2022), we evaluated the prevalence of: being uninsured, not having a primary care doctor, healthcare cost concerns in the past 12 months, and not having a physical exam in the past 12 months.

**Results:**

Among 3,301,491 adults eligible for type 2 diabetes screening, mean age was 52.2 years and 52.3% were women. The prevalence of barriers to preventive care showed substantial heterogeneity among all racial and ethnic groups. Prevalence among multiple race subgroups generally fell in between estimates of their corresponding single race groups. Adults reporting both American Indian/Alaska Native (AIAN) and White race had a higher prevalence of healthcare cost concerns than adults who reported either racial group alone (AIAN and White: 17.8%, 95% confidence interval: [17.0-18.7%]; AIAN: 15.7% [15.0-16.4%]; White: 9.8% [9.7–9.8%]). The opposite pattern was observed among adults who reported both Asian and Pacific Islander race (Asian and Pacific Islander: 6.0% [5.0-7.2%]; Asian: 7.7% [7.3–8.1%]; Pacific Islander: 12.5% [11.4–13.7%]). This latter pattern was also observed for being uninsured among adults who reported both Pacific Islander and White race (Pacific Islander and White: 6.6% [4.9–8.7%]; Pacific Islander: 13.2% [11.9–14.7%]; White: 8.1% [8.0-8.2%]), and those who reported both Asian and White race (Asian and White: 5.2% [4.2–6.5%]; Asian: 6.9% [6.5–7.3%]; White: 8.1% [8.0-8.2%]).

**Conclusions:**

These findings highlight the heterogeneity in preventive care barriers among subgroups of adults of multiple races. Identification of the subgroups at greatest risk may allow for effective and tailored interventions to increase access to preventive care for type 2 diabetes.

**Supplementary Information:**

The online version contains supplementary material available at 10.1186/s12889-025-24692-y.

## Introduction

Disparities in prevalence of type 2 diabetes (T2D) risk factors are well-documented among racial and ethnic groups. However, these are less well described in people of multiple races, a group in the United States projected to grow threefold from 2016 to 2060 [1]. In epidemiologic studies, people of multiple races are frequently aggregated into a single group, combined with other groups, or excluded, despite heterogeneity in T2D risk related to unique underlying patterns of socioeconomic, sociocultural, and other factors that may impact risk factors and health outcomes [2]. Given the complexity of factors that may affect the health of adults who identify with multiple races, there might be a need to investigate health disparities among subgroups of multiple races. Existing studies have demonstrated substantial heterogeneity in risk factors and health outcomes such as obesity and cardiovascular disease (CVD), respectively, among disaggregated groups of multiple races, demonstrating the importance of evaluating disparities using disaggregated data [3, 4].

Much research exists on lifestyle-related risk factors for T2D, whereas less attention has been given to barriers to preventive care, which may impact screening rates and clinical outcomes [5–7]. For example, a common barrier to preventive care is lack of health insurance, which can affect approximately 13% of adults in the US as of 2023, with pronounced disparities across racial and ethnic groups [8]. Lack of insurance may then lead to a greater risk of being unaware of having diabetes [9], as well as delayed care for diabetes [10]. Cost may also be a barrier to healthcare access, with a previous study reporting 7% of adults reporting foregoing care due to cost concerns [11]. Cost-related barriers may also differ across racial and ethnic groups. For example, a greater proportion of Hispanic than White adults forego care due to financial barriers [12]. To our knowledge, prior studies among adults at risk of T2D have not evaluated barriers to preventive care by disaggregated subgroups of multiple races. We therefore evaluated the prevalence of select barriers to preventive care in a large representative sample of US adults eligible for T2D screening by disaggregated subgroups of multiple races.

## Methods

### Study sample

Data were from the Behavioral Risk Factor Surveillance System (BRFSS), an annual random-digit-dialed landline and cellular telephone-based survey of a randomly selected representative sample of noninstitutionalized adults aged ≥ 18 years, from the 50 states, District of Columbia, and participating US territories [13]. BRFSS collects information from respondents on health-related risk factors, healthcare access, and comorbidities. Telephone interviewers obtain verbal informed consent by reading a script and describing the survey and confidentiality of collected information. The present analysis used BRFSS data from 2013, when information on subgroups of multiple races were first collected, to 2022. The analysis included adults eligible for diabetes screening based on guidelines adapted from the American Diabetes Association (ADA) [14] for variables available in each BRFSS survey year (Supplemental Table 1). The ADA guidelines were selected a priori to define the study population, as the guidelines comprise a frequently used set of criteria to define a screening-eligible adult population at high risk of diabetes, and due to the pertinence of barriers to preventive care in obtaining diabetes screening. The construction of the study population is displayed in Supplemental Fig. 1. This activity was reviewed by CDC and was conducted consistent with applicable federal law and CDC policy (See e.g., 45 C.F.R. part 46, 21 C.F.R. part 56; 42 U.S.C. § 241(d); 5 U.S.C. § 552a; 44 U.S.C. § 3501 et seq.). Conduct of the study was performed in accordance with the 1964 Helsinki Declaration and its later amendments or comparable ethical standards. Institutional review board approval was not required as the study only comprised analysis of secondary, deidentified data.

### Race and ethnicity

For the present study, respondents who reported Hispanic ethnicity were categorized as Hispanic regardless of race due to potential high misclassification when combined with race [15, 16]. Non-Hispanic (NH) respondents were categorized by race. Respondents reporting multiple races were categorized into the following groups: American Indian/Alaska Native (AIAN) + Asian, AIAN + Black, AIAN + Pacific Islander, AIAN + White, Asian + Black, Asian + White, Asian + Pacific Islander, Black + Pacific Islander, Black + White, Pacific Islander + White. Respondents reporting three or more races comprised a single category due to small sample sizes. Respondents reporting “Other” race, alone or in combination with another race, comprised the NH Other category.

### Measures

Self-reported sociodemographic variables included age, sex, educational attainment (less than high school, high school, more than high school) and employment status (employed, not employed, retired). Comorbidities diagnosed by a healthcare professional included cardiovascular disease (angina, coronary heart disease, myocardial infarction, or stroke) and gestational diabetes. Lifestyle factors included current smoking and physical inactivity, defined as no leisure-time physical activity in the past month. The BRFSS survey included questionnaire items on four select barriers to preventive care which were categorized as dichotomous variables : (1) being uninsured; (2) not having a primary care doctor; (3) reporting healthcare cost concerns in the past 12 months; and (4) not having a physical exam in the past 12 months. Barriers to preventive care were self-reported by respondents. As the questionnaire on health insurance status substantially changed in 2021, corresponding analyses are conducted on a subset of the total sample from the 2013 through 2020 surveys (*n* = 2,647,321). Questionnaire items used to code these variables are shown in Supplemental Table 2.

### Statistical analysis

The denominator represented the adult population eligible for T2D screening as defined in Supplemental Table 1. Prevalence of each barrier to preventive care and corresponding 95% confidence intervals (CI) were estimated using multivariable logistic models. Two models were evaluated. Model 1 adjusted for age, sex, and survey year. In a secondary analysis, Model 2 further adjusted for education, employment, current smoking, and CVD, to examine the extent to which these variables may explain any disparities observed by racial and ethnic groups in Model 1. Model specification remained parsimonious to ensure reliable estimates due to the small sample sizes in some subgroups of multiple races. Prevalence estimates and corresponding confidence intervals for each subgroup were obtained using the PREDMARG function in SUDAAN software. The PREDMARG function estimates the predictive margins, which represent the sample weighted average of the predicted response (i.e. prevalence of each barrier to preventive care) after adjusting for model covariates [17, 18]. Sample weights and design variables were used to account for the complex survey design. Analyses were conducted using SAS (version 9.4; SAS Institute) and SUDAAN (version 11.0.1; Research Triangle Institute).

## Results

Among 3,301,491 adults eligible for T2D screening, mean age was 52.2 years, 52.3% were women, 60.1% reported greater than a high school education, and 58.2% were employed (Table 1). Sociodemographic characteristics of adults of multiple races varied by subgroup. For example, all adults of multiple races had a mean age of 45.5 years, while among subgroups, mean age ranged from 37.1 years among the small sample reporting Black + Pacific Islander race to 50.2 years among those reporting AIAN + White race. Prevalence of obesity was 37.0% among all adults of multiple races, and ranged from 27.0% for adults reporting AIAN + Asian race to 49.5% among those reporting AIAN + Pacific Islander race (Table 2).

**Table 1:** Characteristics of US adults eligible for T2D screening by race and ethnicity, BRFSS 2013–2022

	Unweighted No. (weighted %)	Age in years, mean (SE)	Women, % (SE)	Educational attainment, % (SE)			Employment status, % (SE)		
				Less than high school	High school	More than high school	Employed	Not employed	Retired
Total	3,301,491 (100)	52.2 (0.02)	52.3 (0.1)	12.9 (0.1)	27.0 (0.1)	60.1 (0.1)	58.2 (0.1)	20.5 (0.1)	21.2 (0.04)
Hispanic	268,104 (17.4)	43.3 (0.1)	49.2 (0.2)	34.6 (0.2)	26.5 (0.2)	38.9 (0.2)	62.8 (0.2)	29.1 (0.2)	8.1 (0.1)
NH AIAN	51,523 (1.1)	48.0 (0.2)	49.2 (0.5)	18.2 (0.4)	33.3 (0.5)	48.5 (0.5)	53.4 (0.5)	32.0 (0.5)	14.6 (0.4)
NH Asian	75,079 (5.5)	44.1 (0.1)	48.3 (0.4)	4.3 (0.2)	15.0 (0.3)	80.6 (0.3)	68.7 (0.4)	21.8 (0.3)	9.5 (0.3)
NH Black	250,622 (12.2)	47.2 (0.1)	54.3 (0.2)	13.0 (0.1)	30.7 (0.2)	56.3 (0.2)	59.5 (0.2)	25.5 (0.2)	15.1 (0.1)
NH Pacific Islander	13,352 (0.24)	42.9 (0.3)	47.6 (1.0)	10.3 (0.6)	33.8 (1.0)	55.9 (1.0)	69.0 (0.9)	22.9 (0.8)	8.1 (0.5)
NH White	2,529,413 (60.9)	56.7 (0.0)	53.3 (0.1)	7.5 (0.0)	27.4 (0.1)	65.1 (0.1)	55.8 (0.1)	16.7 (0.0)	27.5 (0.1)
NH Other	53,703 (1.4)	55.1 (0.1)	46.9 (0.5)	10.6 (0.3)	22.8 (0.4)	66.6 (0.4)	56.6 (0.5)	19.0 (0.4)	24.5 (0.4)
Multiple Races	59,695 (1.4)	45.5 (0.2)	50.6 (0.5)	10.6 (0.4)	26.1 (0.4)	63.6 (0.5)	59.4 (0.5)	27.3 (0.4)	13.3 (0.3)
AIAN + Asian	201 (< 0.01)	45.9 (2.2)	45.8 (6.5)	13.5 (5.0)	25.1 (6.4)	61.4 (6.9)	61.7 (6.6)	23.1 (4.7)	15.2 (6.1)
AIAN + Black	3,409 (0.11)	47.6 (0.6)	52.3 (1.9)	11.1 (1.7)	24.1 (1.5)	64.8 (1.9)	53.9 (1.9)	30.1 (1.9)	16.0 (1.2)
AIAN + Pacific Islander	108 (< 0.01)	45.7 (4.6)	57.2 (16.7)	NA	NA	NA	66.0 (9.0)	28.7 (8.5)	5.3 (2.8)
AIAN + White	27,955 (0.61)	50.2 (0.2)	47.4 (0.7)	14.1 (0.5)	28.5 (0.6)	57.4 (0.7)	51.8 (0.7)	29.6 (0.6)	18.6 (0.5)
Asian + Black	592 (0.03)	40.1 (0.9)	53.6 (3.5)	5.6 (1.7)	21.2 (2.8)	73.2 (3.1)	68.3 (3.4)	24.9 (3.3)	6.8 (1.5)
Asian + White	6,363 (0.18)	40.7 (0.4)	50.0 (1.3)	4.0 (0.7)	17.6 (1.1)	78.4 (1.2)	71.9 (1.3)	22.5 (1.2)	5.5 (0.6)
Asian + Pacific Islander	4,082 (0.06)	44.4 (0.6)	47.4 (2.0)	7.2 (1.0)	26.3 (1.7)	66.5 (1.8)	69.4 (1.8)	19.9 (1.6)	10.7 (1.1)
Black + Pacific Islander	258 (0.01)	37.1 (1.5)	59.3 (7.8)	7.1 (3.2)	40.4 (8.2)	52.6 (7.7)	72.3 (5.0)	22.0 (4.6)	5.7 (2.5)
Black + White	6,993 (0.25)	38.0 (0.4)	58.2 (1.1)	8.1 (0.7)	26.7 (1.0	65.2 (1.1)	66.5 (1.1)	26.7 (1.1)	6.8 (0.6)
Pacific Islander + White	3,301 (0.05)	43.6 (0.7)	48.7 (2.2)	6.5 (1.0)	28.7 (1.9)	64.7 (2.0)	64.9 (2.2)	24.2 (2.2)	10.9 (1.1)
Three or More Races	6,433 (0.12)	49.4 (0.4)	56.8 (1.5)	7.7 (0.7)	22.8 (1.3)	69.6 (1.4)	60.4 (1.5)	25.5 (1.3)	14.1 (1.0)

Data are shown as weighted means and standard error for continuous variables, and as weighted percentages and standard error for categorical variables

Estimates with a relative standard error > 0.30 are considered statistically unreliable and are therefore displayed as “NA”

Abbreviations: *AIAN*, American Indian/Alaska Native, *BRFSS*, Behavioral Risk Factor Surveillance System, *NH* Non-Hispanic

**Table 2:** Clinical and lifestyle characteristics of US adults eligible for type 2 diabetes screening by race and ethnicity, BRFSS 2013–2022

	Unweighted *n* (weighted %)	Overweight, % (SE)	Obesity, % (SE)	Current smoking, % (SE)	Physical inactivity^a^, % (SE)	CVD, % (SE)
Total	3,301,491 (100)	40.5 (0.1)	32.3 (0.1)	15.7 (0.0)	26.3 (0.1)	8.4 (0.0)
Hispanic	268,104 (17.4)	45.5 (0.2)	38.1 (0.2)	12.6 (0.1)	31.1 (0.2)	4.9 (0.1)
NH AIAN	51,523 (1.1)	39.6 (0.5)	38.6 (0.5)	29.4 (0.5)	27.2 (0.5)	11.2 (0.3)
NH Asian	75,079 (5.5)	52.0 (0.4)	23.9 (0.3)	7.9 (0.2)	19.0 (0.3)	3.1 (0.2)
NH Black	250,622 (12.2)	39.6 (0.2)	42.8 (0.2)	18.6 (0.2)	28.3 (0.2)	7.6 (0.1)
NH Pacific Islander	13,352 (0.24)	40.8 (1.1)	39.0 (1.0)	18.2 (0.8)	21.4 (0.8)	5.4 (0.5)
NH White	2,529,413 (60.9)	38.4 (0.1)	29.1 (0.1)	16.2 (0.1)	25.3 (0.1)	9.9 (0.0)
NH Other	53,703 (1.4)	39.7 (0.5)	27.1 (0.4)	16.4 (0.4)	25.8 (0.4)	10.0 (0.3)
Multiple Races	59,695 (1.4)	41.2 (0.5)	37.0 (0.5)	23.6 (0.4)	21.3 (0.4)	9.8 (9.3)
AIAN + Asian	201 (< 0.01)	45.4 (6.9)	27.0 (7.4)	20.2 (5.3)	19.3 (5.2)	8.7 (3.1)
AIAN + Black	3,409 (0.11)	41.1 (2.1)	39.4 (1.8)	23.3 (1.5)	22.8 (1.4)	13.1 (1.7)
AIAN + Pacific Islander	108 (< 0.01)	39.7 (10.3)	49.5 (11.1)	44.1 (11.7)	NA	4.8 (2.6)
AIAN + White	27,955 (0.61)	38.8 (0.7)	37.0 (0.6)	29.6 (0.6)	24.1 (0.6)	13.9 (0.4)
Asian + Black	592 (0.03)	45.2 (3.6)	35.9 (3.5)	14.3 (2.3)	19.0 (2.8)	5.2 (1.8)
Asian + White	6,363 (0.18)	44.3 (1.4)	29.2 (1.3)	13.7 (1.0)	13.9 (0.9)	3.7 (0.6)
Asian + Pacific Islander	4,082 (0.06)	42.3 (2.0)	31.5 (1.8)	11.6 (0.9)	18.5 (1.5)	4.8 (0.7)
Black + Pacific Islander	258 (0.01)	43.1 (7.4)	43.6 (8.1)	11.1 (3.0)	NA	0.8 (0.3)
Black + White	6,993 (0.25)	42.9 (1.1)	42.9 (1.1)	21.1 (0.9)	19.7 (0.9)	5.1 (0.5)
Pacific Islander + White	3,301 (0.05)	46.6 (2.2)	36.0 (2.0)	19.9 (2.0)	20.0 (1.8)	6.7 (1.2)
Three or More Races	6,433 (0.12)	36.6 (1.5)	37.2 (1.5)	23.7 (1.2)	26.6 (1.2)	11.1 (0.9)

Data are shown as weighted percentages and standard errors

Estimates with a relative standard error > 0.30 are considered statistically unreliable and are therefore displayed as “NA”

Abbreviations: *AIAN* American Indian/Alaska Native, *BRFSS* Behavioral Risk Factor Surveillance System, *CVD* Cardiovascular disease, *NA* Not available, *NH* Non-Hispanic, *SE* standard error

^a^Defined as no leisure-time physical activity in the past month

**Fig. 1:**
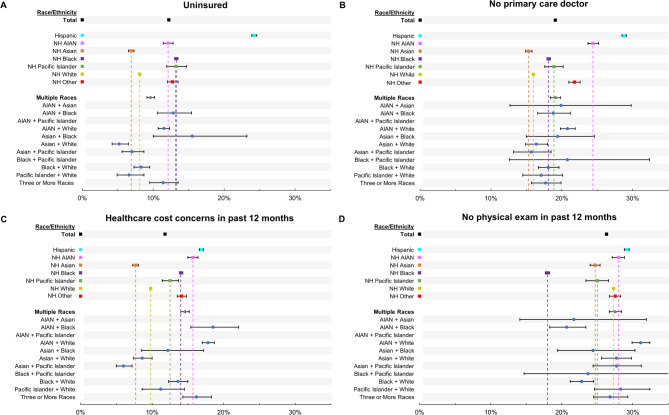
Prevalence of Preventive Care Barriers in US Adults by Race and Ethnicity, BRFSS 2013–2022 (Model 1) Forest plots show the adjusted prevalence of each barrier to preventive care, by race and ethnicity, including subgroups of adults of multiple races. Prevalence is adjusted for age, sex and survey year. Error bars denote 95% confidence intervals. Empty rows indicate estimates with a relative standard error > 0.30 and were therefore considered statistically unreliable. Prevalence estimates for being uninsured are based on a subset of the data from 2013–2020

Prevalence of being uninsured was 12.2% overall (95% CI: 12.1–12.3%), and highest among Hispanic adults at 24.2% (95% CI: 23.9–24.6%) (Fig. 1A, Supplemental Table 3). Among adults of multiple races, the highest prevalence of being uninsured was observed among those reporting AIAN + White race (11.5%, 95% CI: 10.7–12.3%), AIAN + Black race (12.8%, 95% CI: 10.6–15.4%), and Asian + Black race (15.5%, 95% CI: 10.0-23.2%), although the latter estimate had high variability. Adults reporting Pacific Islander + White race or Asian + White race, were less likely to be uninsured than the corresponding single race groups.

A similar pattern was observed for having no primary care doctor (Fig. 1B, Supplemental Table 3). Overall prevalence was 19.1% (95% CI: 19.0-19.2%), with Hispanic adults showing the highest prevalence of 28.8% (95% CI: 28.5–29.1%). Prevalence estimates for adults of multiple races was lowest 15.7% (95% CI: 13.2–18.5%) for Asian + Pacific Islander adults. Prevalence was highest at 20.8% (95% CI: 19.8–21.9%) among AIAN + White adults and 20.8% (95% CI: 12.6–32.4%) among Black + PI adults. Estimates showed high variability among subgroups with smaller samples, such as adults reporting Black + Pacific Islander race or AIAN + Asian race.

For healthcare cost concerns in the past 12 months, overall prevalence was 11.8% (95% CI: 11.7–11.9) (Fig. 1C, Supplemental Table 3). Among adults reporting a single race or ethnicity, Hispanic and NH AIAN adults showed the highest prevalence at approximately 16–17%. Concerns about healthcare cost were more frequent among adults reporting AIAN + White race than those of their component racial groups, while adults reporting Asian + Pacific Islander race showed the opposite pattern.

Overall, 26.3% (95% CI: 26.2–26.4%) of adults reported no physical exam in the past 12 months (Fig. 1D, Supplemental Table 3). Across all groups, prevalence of no physical exam in the past 12 months was generally higher compared with other barriers to preventive care, though variability in estimates was high for subgroups with smaller samples. Among people of multiple races, prevalence ranged from 20.7% (95% CI: 18.3–23.4%) among AIAN + Black adults to 31.1% (95% CI: 29.9–32.4%) among AIAN + White adults.

Supplementary Tables 4 and Supplementary Fig. 2 show results from secondary analyses further adjusting for education, employment, smoking and CVD. Overall, there was reduced heterogeneity in the prevalence of each barrier to preventive care among aggregated racial and ethnic groups. Among subgroups of multiple races, the attenuation in the heterogeneity of prevalence estimates was more limited, and high variability for subgroups with smaller samples persisted.

## Discussion

Among US adults of multiple races eligible for T2D screening, the prevalence of select barriers to preventive care was mostly similar to their component racial groups. In contrast, adults reporting Asian and either Pacific Islander or White race had a lower prevalence of some barriers to preventive care, compared with adults reporting any of the component racial groups alone. Adults reporting AIAN and White race had a higher prevalence of some barriers to preventive care compared with each component racial group. Among adults reporting a single race or ethnicity, Hispanic and NH AIAN adults generally showed the highest prevalence of barriers to preventive care, and NH Asian adults generally showed the lowest prevalence.

While other studies have not investigated barriers to preventive care among adults of multiple races, similar studies have investigated other diabetes-related risk factors or outcomes with varied findings [3, 4, 19]. The most recent study, using electronic health record (EHR) data of insured adults residing in California and Hawaii, showed that adults of multiple races had a higher prevalence of CVD compared with their component racial groups [4]. The different findings in these studies compared with the current study may be due to different categorizations for multiple races, different risk factors and outcomes, and inclusion of only regional populations. In addition, using EHRs to ascertain race may result in greater misclassification compared with self-reported survey data [20].

Given the lack of prior research and the complex combination of individual, interpersonal, community and structural factors [2] that collectively affect health outcomes and may also affect barriers to preventive care among adults of multiple races, potential explanations of our findings may be difficult to assess. In the current study, after adjustment for education, employment, current smoking status and CVD, results changed slightly, suggesting other related factors may better explain the observed heterogeneity in prevalence of barriers to preventive care. Regardless of these uncertainties, we propose hypothetical explanations for some of the findings. For example, some adults of multiple races had a prevalence of some barriers to preventive care that was below the prevalence of their component racial groups, such as among adults reporting Asian + Pacific Islander race. This may be attributed to the large heterogeneity in healthcare-related risk factors among Asian subgroups. For example, in our previous work in the BRFSS population, the prevalence of healthcare cost concerns ranged from 5.7% among Chinese adults to 11.1% among Korean adults [21]. Our observed findings may therefore be explained if adults reporting only NH Asian race have a greater proportion of specific subgroups with a high prevalence of healthcare cost concerns, compared with adults reporting Asian + Pacific Islander race. Additionally, these findings may also be attributed to regional differences in socioeconomic and other determinants of healthcare barriers if adults reporting Asian + White or Asian + Pacific Islander race are more likely to reside in certain regions of the US compared with individuals identifying with their component racial groups.

The current study showed a higher prevalence of no recent physical exam and no primary care doctor than being uninsured or having cost concerns, suggesting that non-financial barriers may be underlying some of the barriers in preventive care. For example, some adults may forego or delay care due to patient-level barriers such as family and work commitments, as well as provider-related barriers such as discrimination and lack of cultural or linguistic competency [22, 23]. For adults of multiple races, identifying and addressing the latter may be more complex than for those of single race or Hispanic ethnicity. Information on self-identification with multiple races, rather than observer-reported race or an aggregated multiple race group, may be needed for effective, culturally-tailored interventions to increase access to preventive care. While such interventions may be effective in preventing type 2 diabetes by promoting healthy lifestyle behaviors [24, 25], less research exists on interventions’ effectiveness to address barriers to preventive care such as difficulty in navigating the healthcare system or lack of knowledge of health insurance options, regardless of identification with multiple races. Addressing linguistic barriers by using professional interpreters may provide accurate translation and cultural insight, potentially increasing use of preventive care [26, 27]. However, successful implementation can be limited by lack of provider training [26, 27]. Lastly, effective prevention and screening for diabetes may be better informed by a comprehensive risk profile from the patient, collecting information on language preferences, psychosocial factors, cultural sensitivities, use of traditional medicine, and other factors that characterize the unique profile of each patient who identifies with multiple races [28].

The substantial heterogeneity in prevalence of barriers to preventive care observed in our study suggests a need for data collection methods that accurately record multiple races. Standardized categories for subgroups of multiple races might be needed for more accurate comparison among different studies. In particular, the two-question approach in recording race and ethnicity as separate constructs may lead to misclassification among Hispanic adults. In response to a questionnaire item inquiring on racial identity, prior evidence suggests that the majority of Hispanic adults may indicate an “other” category, White race, or skip the question [15, 16]. Substantial misclassification may therefore result when categorizing individuals who identify with both a Hispanic ethnicity and a racial group. As the current study showed the aggregated Hispanic group had the highest prevalence of being uninsured and not having a primary care doctor, accurate classification of adults who identify as Hispanic and one or more races may therefore reveal how these prevalence estimates differ when disaggregated. As such, a 2024 update to the Office of Management and Budget’s standards for race and ethnicity data [29] may facilitate more accurate future data collection for subgroups of multiple races.

With increasing use of data sources such as EHRs and insurance claims in health research, healthcare systems may need to ensure accurate recording of information on multiple races [30]. Methods may include the use of platforms that allow selection of multiple races and use of self-reported rather than observer-reported race. Prior studies show generally good agreement between self-reported and observer-reported race among adults reporting Black or White race, but poor agreement among other groups including those reporting multiple races [31]. Therefore, a substantial proportion of race data in administrative datasets may be misclassified, particularly affecting adults who self-identify with multiple races.

The study is subject to at least five limitations. First, preventive care barriers evaluated in this study were limited to the four items included in the questionnaire. Second, preventive care barriers, were based on self-reported responses which may be subject to recall or social desirability bias. Third, adults of multiple races may have been undercaptured as they comprised 1.3% of the study population, compared to the US Census data estimate of 2.1% [32] over a similar time period. Fourth, we were not able to evaluate adults reporting both Hispanic ethnicity and a racial group due to potentially high misclassification. Lastly, small sample sizes for some subgroups of multiple races resulted in prevalence estimates with high variability, were suppressed, or were aggregated, such as for adults who reported three or more races. Further, small sample sizes in subgroups did not allow for evaluating trends over time, which may identify which multiple race groups have the most rapidly increasing prevalence of barriers to preventive care.

## Conclusions

With the rapid growth of the adult population reporting more than one race, the need to better identify people of multiple races may also grow to address potential inequities in access to preventive care. Due to a lack of current research, potential strategies to address inequities may require additional studies using standardized data on subgroups of multiple races. Such evidence may guide future public health policy and clinical practice efforts, including the accurate design and implementation of culturally tailored interventions for the prevention of type 2 diabetes and other diseases in adults of multiple races.

## Supplementary Information

Supplementary Material 1.

## Data Availability

The data that support the findings of this study are available from: https://www.cdc.gov/brfss/annual_data/annual_data.htm. Data on disaggregated race and ethnicity categories are not available per terms of the data use agreement with the Division of Population Health, Centers for Disease Control and Prevention.

## Abbreviations

AIAN: American indian/alaska native
BRFSS: Behavioral risk factor surveillance system
NA: Not available
NH: Non-Hispanic
SE: standard error
T2D: Type 2 Diabetes
