# Supplementary material for: Barriers to preventive care among US adults of multiple races eligible for type 2 diabetes screening: an observational study
Source: BMC Public Health. 2026 Jan 10;26:504. doi: 10.1186/s12889-025-24692-y (PMC12882277; doi:10.1186/s12889-025-24692-y)

**Supplemental Data**

Barriers to Preventive Care Among US Adults of Multiple Races Eligible for Type 2 Diabetes Screening

**Supplemental Table 1.** Diabetes Screening Eligibility Criteria Used for Study Inclusion (Adapted from American Diabetes Association Guidelines)

| 1. Does not currently have diabetes 2. Adults with overweight or obesity (body mass index ≥25 kg/m^2^ or ≥23 kg/m^2^ in non-Hispanic Asian respondents) who have one or more of the following risk factors:    1. Race or ethnicity: Hispanic, Black, American Indian/Alaska Native, Asian, Pacific Islander    2. History of cardiovascular disease    3. Physical inactivity 3. History of gestational diabetes 4. Age 35 or older |
| --- |

Note: Hypertension and hypercholesterolemia are included in the American Diabetes Association guidelines, but were not used for inclusion criteria since data for these variables were collected in the survey biennially rather than annually.

**Supplemental Table 2.** Questionnaire Items and Responses Used to Measure Risk Factors

| **Smoking**  “Yes” to: Have you smoked at least 100 cigarettes in your entire life?  AND  “Every day” or “Some days” to: Do you now smoke cigarettes every day, some days, or not at all?  **Physical inactivity**  “No” to: During the past month, other than your regular job, did you participate in any physical activities or exercises such as running, calisthenics, golf, gardening, or walking for exercise?  **Uninsured^a^**  2013–2020: A response of “No” to: Do you have any kind of health care coverage, including health insurance, prepaid plans such as HMOs, or government plans such as Medicare, or Indian Health Service? 2021–2022: A response of “No coverage of any type” to: What is the current primary source of your health insurance?  **No primary care doctor**  A response of “No” to:  2013–2020: Do you have one person you think of as your personal doctor or health care provider?  2021–2022: Do you have one person or a group of doctors that you think of as your personal health care provider?  **Healthcare cost concerns in the past 12 months**  A response of “Yes” to:  2013–2020: Was there a time in the past 12 months when you needed to see a doctor but could not because of cost?  2021–2022: Was there a time in the past 12 months when you needed to see a doctor but could not because you could not afford it?  **No physical exam in past 12 months**  2013–2022: Any of the following affirmative response to the question, “About how long has it been since you last visited a doctor for a routine checkup? [A routine checkup is a general physical exam, not an exam for a specific injury, illness, or condition.]  “Within the past 2 years (1 year but less than 2 years ago)” or  “Within the past 5 years (2 years but less than 5 years ago)” or  “5 or more years ago” or  “Never”. |
| --- |

^a^Responses from 2021-2022 were not used due to substantial changes in the questionnaire wording and corresponding responses

**Supplemental Table 3.** Adjusted Prevalence of Barriers to Preventive Care in US Adults Without Type 2 Diabetes by Racial and Ethnic Subgroups, Behavioral Risk Factor Surveillance System 2013–2022 (Model 1)

|  | **Prevalence (95% confidence interval)** | | | |
| --- | --- | --- | --- | --- |
|  | **Uninsured**^a^ | **No primary care doctor^b^** | **Healthcare cost concerns in past 12 months** **^c^** | **No physical exam in past 12 months^d^** |
| Total | 12.2 (12.1-12.3) | 19.1 (19.0-19.2) | 11.8 (11.7-11.9) | 26.3 (26.2-26.4) |
|  |  |  |  |  |
| Hispanic | 24.2 (23.9-24.6) | 28.8 (28.5-29.1) | 16.9 (16.6-17.2) | 29.2 (28.8-29.5) |
| NH AIAN | 12.1 (11.4-12.8) | 24.4 (23.7-25.2) | 15.7 (15.0-16.4) | 28.0 (27.1-28.8) |
| NH Asian | 6.9 (6.5-7.3) | 15.3 (14.9-15.8) | 7.7 (7.3-8.1) | 24.7 (24.0-25.4) |
| NH Black | 13.2 (13.0-13.5) | 18.1 (17.9-18.4) | 14.0 (13.8-14.3) | 18.0 (17.7-18.3) |
| NH Pacific Islander | 13.2 (11.9-14.7) | 18.9 (17.6-20.2) | 12.5 (11.4-13.7) | 25.0 (23.4-26.6) |
| NH White | 8.1 (8.0-8.2) | 16.0 (15.9-16.1) | 9.8 (9.7-9.8) | 27.3 (27.2-27.4) |
| NH Other | 12.7 (12.0-13.5) | 21.8 (21.0-22.6) | 14.1 (13.5-14.8) | 27.5 (26.7-28.3) |
|  |  |  |  |  |
| Multiple Races | 9.6 (9.1-10.2) | 19.1 (18.4-19.8) | 14.6 (14.0-15.2) | 27.5 (26.7-28.4) |
| AIAN + Asian | NA | 19.9 (12.7-29.8) | NA | 21.7 (14.1-31.9) |
| AIAN + Black | 12.8 (10.6-15.4) | 18.8 (16.6-21.2) | 18.5 (15.4-22.1) | 20.7 (18.3-23.4) |
| AIAN + Pacific Islander | NA | NA | NA | NA |
| AIAN + White | 11.5 (10.7-12.3) | 20.8 (19.8-21.9) | 17.8 (17.0-18.7) | 31.1 (29.9-32.4) |
| Asian + Black | 15.5 (10.0-23.2) | 19.4 (15.0-24.6) | 12.2 (8.5-17.2) | 24.4 (19.4-30.3) |
| Asian + White | 5.2 (4.2-6.5) | 16.4 (14.9-18.0) | 8.6 (7.4-10.0) | 27.7 (25.6-29.8) |
| Asian + Pacific Islander | 7.0 (5.6-8.7) | 15.7 (13.2-18.5) | 6.0 (5.0-7.2) | 27.7 (24.4-31.2) |
| Black + Pacific Islander | NA | 20.8 (12.6-32.4) | NA | 23.7 (14.7-35.9) |
| Black + White | 8.3 (7.3-9.5) | 18.1 (16.7-19.6) | 13.6 (12.3-15.0) | 22.8 (21.2-24.5) |
| Pacific Islander + White | 6.6 (4.9-8.7) | 17.1 (14.5-20.1) | 11.2 (8.6-14.5) | 28.3 (24.6-32.4) |
| Three or More Races | 11.4 (9.5-13.5) | 17.7 (15.7-19.9) | 16.2 (14.3-18.3) | 26.8 (24.5-29.3) |

Notes: Estimates with a relative standard error > 0.30 are considered statistically unreliable and are therefore shown as “NA.”

Prevalence is adjusted for age, sex and survey year. Prevalence estimates for being uninsured are based on a subset of the data from 2013-2020.

Abbreviations: AIAN, American Indian/Alaska Native; NA, Not available; NH, Non-Hispanic

^a^Having any kind of healthcare coverage, including private or public plans.

^b^Having one person or a group of doctors considered as their personal healthcare provider(s).

^c^Reported a time in the past 12 months when a doctor could not be seen due to cost.

^d^Reported seeing a doctor for a general physical exam within the past 12 months.

**Supplemental Table 4.** Adjusted Prevalence of Barriers to Preventive Care in US Adults Without Type 2 Diabetes by Racial and Ethnic Subgroups, Behavioral Risk Factor Surveillance System 2013–2022 (Model 2)

|  | **Prevalence (95% confidence interval)** | | | |
| --- | --- | --- | --- | --- |
|  | **Uninsured**^a^ | **No primary care doctor^b^** | **Healthcare cost concerns in past 12 months** **^c^** | **No physical exam in past 12 months^d^** |
| Total | 12.0 (11.9-12.1 ) | 18.9 (18.8-19.0 ) | 11.7 (11.6-11.8 ) | 26.4 (26.2-26.5 ) |
|  |  |  |  |  |
| Hispanic | 19.1 (18.8-19.4 ) | 25.4 (25.1-25.7 ) | 15.3 (15.0-15.5 ) | 28.3 (27.9-28.6 ) |
| NH AIAN | 9.7 (9.1-10.3 ) | 22.4 (21.7-23.2 ) | 13.3 (12.6-13.9 ) | 27.4(26.6-28.3 ) |
| NH Asian | 8.7 (8.3-9.2 ) | 17.2 (16.7-17.7 ) | 8.8 (8.4-9.3 ) | 25.9 (25.2-26.6 ) |
| NH Black | 12.7 (12.4-13.0 ) | 17.9 (17.6-18.1 ) | 13.5 (13.3-13.8 ) | 18.2 (17.9-18.5 ) |
| NH Pacific Islander | 12.6 (11.3-14.0 ) | 18.3 (17.0-19.6 ) | 11.8 (10.7-13.0 ) | 25.3 (23.7-26.9 ) |
| NH White | 8.9 (8.7-9.0 ) | 16.5 (16.4-16.7 ) | 10.0 (9.9-10.1 ) | 27.5 (27.4-27.7 ) |
| NH Other | 12.7 (12.0-13.5 ) | 22.2 (21.4-23.2 ) | 14.3 (13.6-15.1 ) | 27.7 (26.8-28.6 ) |
|  |  |  |  |  |
| Multiple Races | 9.7 (9.2-10.3 ) | 19.0 (18.4-19.6 ) | 13.8 (13.2-14.4 ) | 27.7 (27.0-28.5 ) |
| AIAN + Asian | NA | 19.2 (11.1-31.1 ) | NA | 21.4 (13.4-32.4 ) |
| AIAN + Black | 12.8 (10.4-15.7 ) | 19.5 (17.1-22.1 ) | 16.6 (13.9-19.6 ) | 21.6 (19.0-24.4 ) |
| AIAN + Pacific Islander | NA | NA | NA | NA |
| AIAN + White | 10.1 (9.4-10.9 ) | 19.8 (18.8-20.9 ) | 15.2 (14.4-16.1 ) | 30.7 (29.5-31.9 ) |
| Asian + Black | 17.1 (10.9-25.7 ) | 22.2 (17.3-28.0 ) | 12.5 (8.7-17.8 ) | 24.9 (19.6-31.1 ) |
| Asian + White | 6.8 (5.4-8.4 ) | 18.2 (16.6-19.9 ) | 9.5 (8.1-11.1 ) | 29.1 (27.0-31.3 ) |
| Asian + Pacific Islander | 7.7 (6.2-9.6 ) | 16.3 (13.7-19.2 ) | 6.6 (5.5-8.0 ) | 28.7 (25.3-32.3 ) |
| Black + Pacific Islander | NA | 21.6 (14.2-31.4 ) | 14.2 (7.9-24.2 ) | 26.9 (18.9-36.9 ) |
| Black + White | 8.8 (7.7-10.1 ) | 18.5 (17.0-20.0 ) | 13.8 (12.4-15.4 ) | 23.3 (21.6-25.0 ) |
| Pacific Islander + White | 7.1 (5.4-9.2 ) | 18.1 (15.3-21.3 ) | 11.5 (8.9-14.7 ) | 29.0 (25.2-33.2 ) |
| Three or More Races | 11.4 (9.5-13.5 ) | 17.9 (15.8-20.2 ) | 15.0 (13.2-16.9 ) | 27.0 (24.6-29.6 ) |

Notes: Estimates with a relative standard error > 0.30 are considered statistically unreliable and are therefore shown as “NA”.

Prevalence is adjusted for age, sex survey year, education, employment status and cardiovascular disease. Prevalence estimates for being uninsured are based on a subset of the data from 2013-2020.

Abbreviations: AIAN, American Indian/Alaska Native; NA, Not available; NH, Non-Hispanic.

^a^Having any kind of healthcare coverage, including private or public plans.

^b^Having one person or a group of doctors considered as their personal healthcare provider(s).

^c^Reported a time in the past 12 months when a doctor could not be seen due to cost.

^d^Reported seeing a doctor for a general physical exam within the past 12 months.

**Supplemental Figure 1.** Construction of Study Population for Adults Eligible for Type 2 Diabetes Screening, Behavioral Risk Factor Surveillance System 2013–2022

**
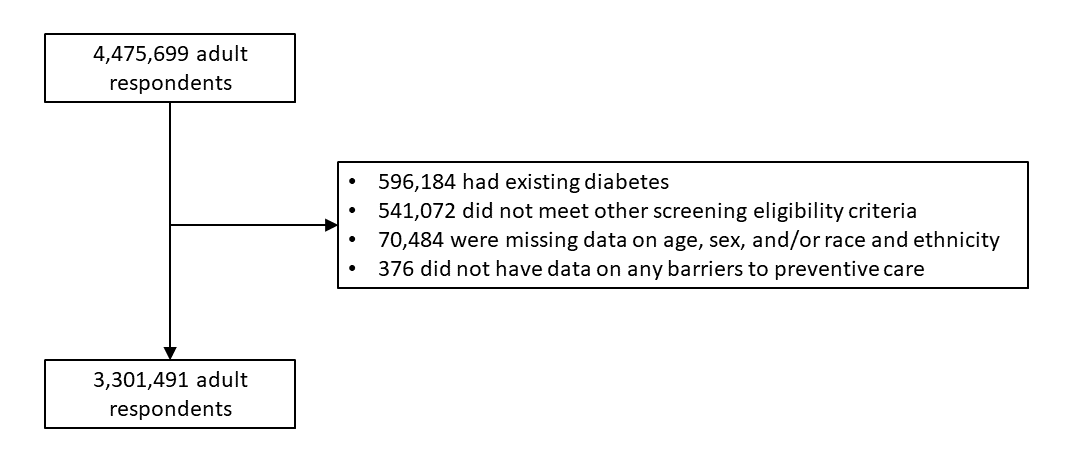
**

**Supplemental Figure 2** Prevalence of Preventive Care Barriers in US Adults by Race and Ethnicity, BRFSS 2013–2022, Model 2

Forest plots show the adjusted prevalence of each barrier to preventive care, by race and ethnicity, including subgroups of adults of multiple races. Prevalence is adjusted for age, sex, survey year, education, employment status, current smoking, cardiovascular disease. Error bars denote 95% confidence intervals. Empty rows indicate estimates with a relative standard error > 0.30 and were therefore considered statistically unreliable. Prevalence estimates for being uninsured are based on a subset of the data from 2013-2020.

Abbreviations: AIAN, American Indian/Alaska Native; BRFSS, Behavioral Risk Factor Surveillance System; NH, Non-Hispanic.


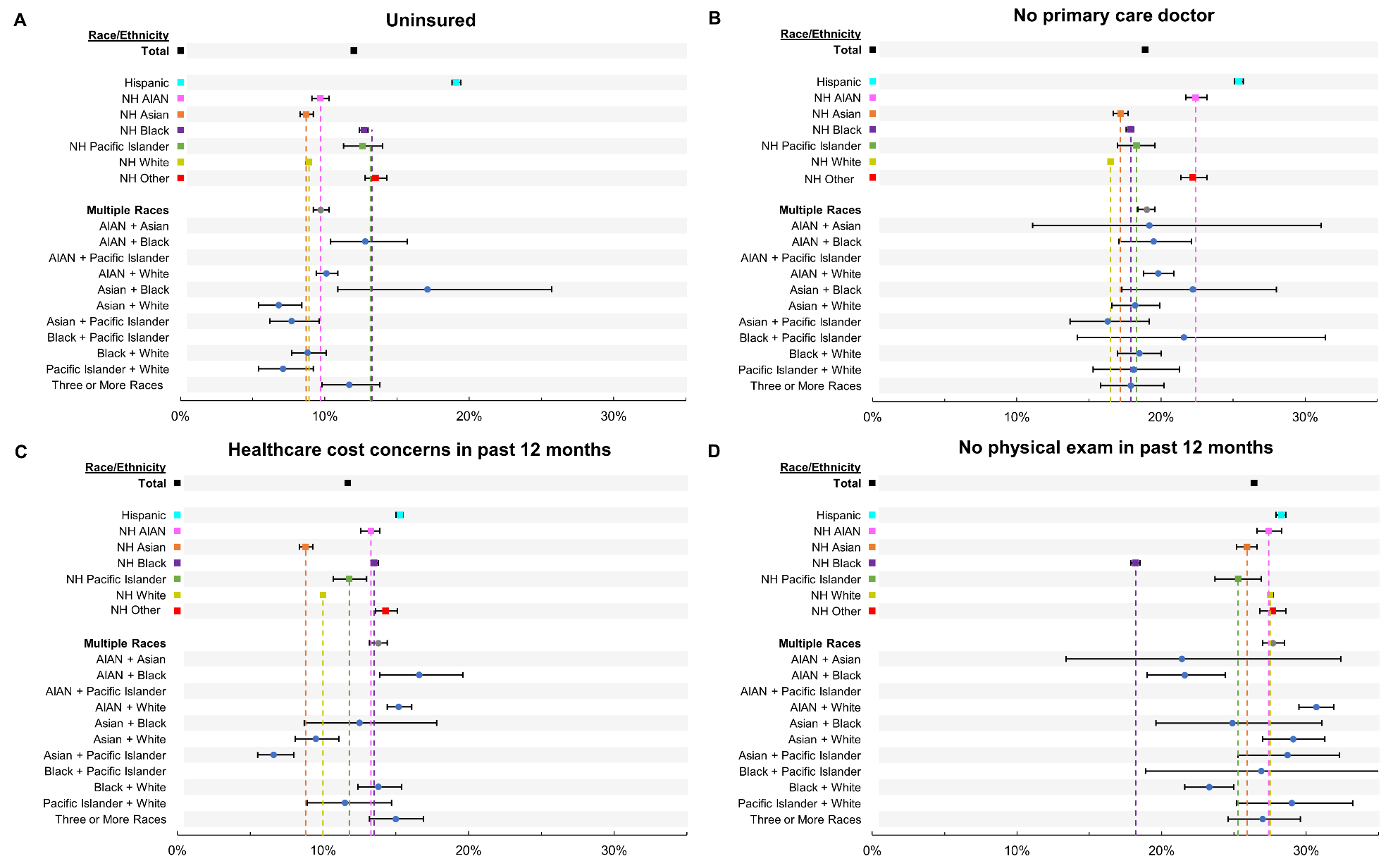

Supplement: Supplementary file 1 — Supplementary Material 1. [file 12889_2025_24692_MOESM1_ESM.docx]
